# Circ-MMP2 (circ-0039411) induced by FOXM1 promotes the proliferation and migration of lung adenocarcinoma cells in vitro and in vivo

**DOI:** 10.1038/s41419-020-2628-4

**Published:** 2020-06-08

**Authors:** Xin Lv, Hongping Huang, Hui Feng, Zhonghua Wei

**Affiliations:** 1 Department of Respiration, Linyi People’s Hospital, Linyi, 276000 Shandong China; 2 Department of Eastern Respiratory and Critical Care Medicine, Linyi People’s Hospital, Linyi, 276034 Shandong China; 3 Linyi People’s Hospital Office, Linyi, 276000 Shandong China; 4 Department of Eastern General Internal Medicine, Linyi People’s Hospital, Linyi, 276034 Shandong China

**Keywords:** Cancer stem cells, Lung cancer

## Abstract

Numerous reports have stated the significance of cellular events such as proliferation, migration and EMT (epithelial-mesenchymal transition) for cancer development, but the related molecular mechanism remains elusive. FOXM1 (forkhead box transcription M1) is a nuclear co-activator participating in lung adenocarcinoma (LUAD). Thus, this study tried to explain the function of FOXM1 and its downstream molecular mechanism in LUAD. We uncovered FOXM1 upregulation in LUAD and demonstrated that FOXM1 facilitated β-catenin nuclear translocation to activate the transcription of downstream genes. Moreover, we discovered that FOXM1 transcriptionally activated circ0039411 which derived from matrix metallopeptidase 2 (MMP2) (also named as circ-MMP2), while MMP2 is a known downstream target of β-catenin. As for functional investigation, knockdown of circ-0039411 suppressed the proliferation, migration and EMT in LUAD cells and also hindered in vivo growth and metastasis of LUAD tumor. Mechanistically, circ-0039411 enhanced the stability of FOXM1 mRNA by recruiting IGF2BP3 (insulin like growth factor 2 mRNA binding protein 3), thus forming a positive feedback loop. In conclusion, this study revealed that FOXM1-induced circ-MMP2 (circ-0039411) contributes to malignant behaviors of LUAD cells via relying on FOXM1, potentially infusing inspirations for the search of new molecular targets for LUAD treatment.

## Introduction

Lung cancer belongs to a kind of primary cause of cancer-induced deaths in the world. There was at least 1.6 million patients confirmed as lung cancer and not less than 1.5 million people died from lung cancer around the world in 2012^1^. Lung adenocarcinoma (LUAD) is a common subtype of lung cancer^2^. Even though there are many improvements in the treatment of LUAD, the 5-year survival rate of LUAD patient is still poor^3^. Patients with LUAD usually lack obvious clinical symptoms, which seriously delays the diagnosis and treatment of LUAD and leads to dim chance accordingly for them to receive useful LUAD treatment. Hence, it is very critical to research mechanisms related to LUAD for searching more biomarkers and developing novel treatments.

FOXM1, a winged-helix transcription factor^4^, is recognized as a modulator of the cell-cycle progression through regulating the associated genes including p27Kip1, p21Cip1, and Cdc25A/B^5,6^. Association of FOXM1 with carcinogenesis has been supported by strong evidences. Previously, studies have argued that besides cell cycle, FOXM1 can also influence many other cancer-related processes, like cellular growth, invasion, angiogenesis, metastasis, and EMT^7–9^. Researches have shown the participation of FOXM1 in gastric cancer^10^, bladder cancer^11^, and cervical cancer^12^. Importantly, several reports have established the link between FOXM1 and LUAD. For example, non-coding RNA PTTG3P recruited FOXM1 to trigger BUB1B transcription, aggravate anaphase transition of mitosis and strengthen cisplatin/paclitaxel resistance in LUAD cells^13^. FOXM1 has also been revealed to serve as a contributing factor of EMT and metastasis in LUAD cells by trans-activating SNAIL and mediating the effect of TGF-β1^14,15^. Nevertheless, deeper understanding of mechanisms relating to FOXM1 is still required.

Circular RNAs (circRNAs) have been reported as a new group of non-coding RNAs^16^. More than 30000 circRNAs have been detected by sequencing and computational methods^17^. As discovered by recent studies, circRNAs can participate in many biological processes of cancers^18^. For example, circ-ABCB10 enhances breast cancer cell growth by sponging miR-1271^19^. Circ-0020397 modulates the progression of colorectal cancer cells via regulating the expression of TERT and PD-L1^20^. Intriguingly, several circRNAs are supported to function in cancers via regulating FOXM1. For instance, circ-HIPK3 sequesters miR-149 to activate FOXM1 in non-small cell lung cancer^21^. Also, circTP63 induces FOXM1 level in lung squamous cell carcinoma^22^. FOXM1 is proved to regulate Wnt/β-catenin, a well-known carcinogenic pathway in cancers, by interacting with β-catenin and facilitating its nuclear import^23–25^. Therefore, we are interested in whether FOXM1 could affect the circRNA form of downstream target genes of β-catenin. There are several key downstream target genes of β-catenin, such as CDK1 (hsa_circ_000577, hsa_circ_0093827), SOX2 (hsa_circ_0122884), MYC (hsa_circ_0085533, hsa_circ_0085534, hsa_circ_0085535) and MMP2 (hsa_circ_0039407, hsa_circ_0039408, hsa_circ_0039409, hsa_circ_0039410, hsa_circ_0039411, hsa_circ_0105604). Meanwhile, circ-0039411 (the circRNA annotated to MMP2) has been reported to play the oncogenic role in papillary thyroid cancer^26^. However, we knew few about whether circ-0039411 participated in the progression of LUAD.

Hence, in this study, we sought to search the impact of FOXM1 on circ-MMP2 (circ-0039411) and the influence of FOXM1/circ-MMP2 on the development of LUAD.

## Results

### Silencing FOXM1 abrogated cell proliferation, migration, and EMT in LUAD cells and restrained LUAD tumor growth and metastasis in vivo

**Fig. 1:**
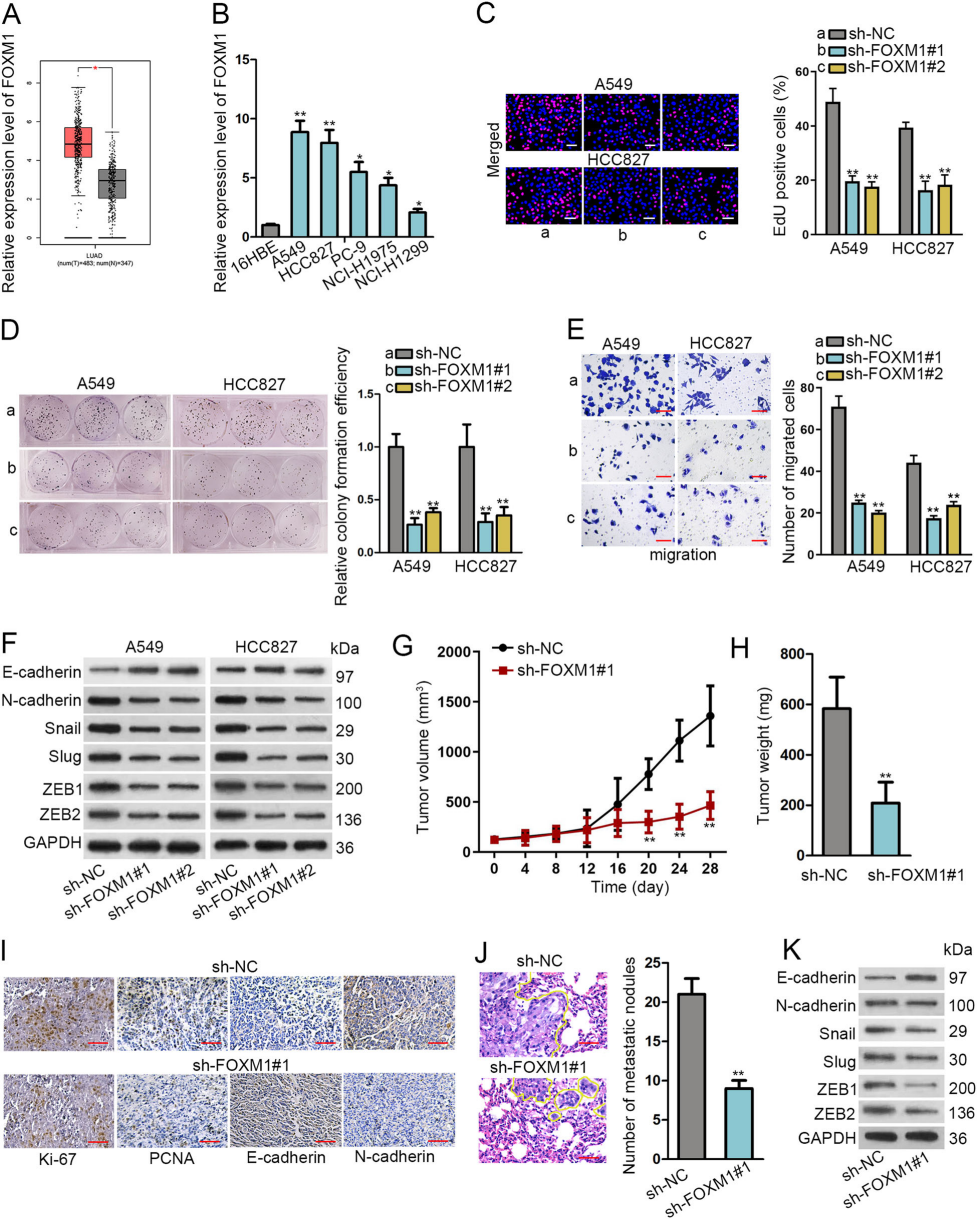
FOXM1 promoted cell proliferation, migration, EMT as well as tumor growth and metastasis in LUAD. **a** FOXM1 level in LUAD samples versus normal ones were obtained from TCGA. **b** qRT-PCR results of FOXM1 level in LUAD cells versus normal ones. **c** Pictures of EdU-stained cells (scale bar = 100 μm) was taken and ratio of EdU-stain ratio was evaluated. **d** Images of colonies and quantification of colony formation efficiency in LUAD cells under FOXM1 knockdown. **e** Migrated cells in transwell chamber were pictured and counted under FOXM1 knockdown (scale bar = 100 μm). **f** Western blot was adopted to detect EMT-related markers. GAPDH was an internal control. **g**, **h** Tumor volume and weight was tested after silencing FOXM1. **i** IHC was utilized to test cell proliferation and EMT markers after silencing FOXM1 (scale bar = 50 μm). **j** Number of metastatic nodules was detected by H&E staining (scale bar = 50 μm). **k** Western blots of EMT-related proteins in xenografts with FOXM1 knockdown. *P < 0.05, **P < 0.01.

First, we tried to comprehend the role of FOXM1 in LUAD. The significantly high FOXM1 expression in LUAD samples (*n* = 483) versus normal ones (*n* = 347) was obtained from a public TCGA database (Fig. 1a). Afterwards, qRT-PCR confirmed the higher expression of FOXM1 in LUAD cells (A549, HCC827, PC-9, NCI-H1975 and NCI-H1299) than that in normal 16HBE cells (Fig. 1b), and two cell lines (A549 and HCC827) expressing the highest FOXM1 level were chosen for later use. The satisfactory knockdown efficiency of FOXM1 was verified in A549 and HCC827 cells with the transfection of sh-FOXM1#1/#2 compared to those with sh-NC control (Supplementary Fig. S1A).

Next, FOXM1 function was interrogated via loss-of-function assays. As observed, FOXM1 knockdown decreased the proportion of EdU-stained proliferative cells and lowered the colony formation efficiency (Fig. 1c, d). Using a transwell system, we monitored that FOXM1 knockdown repressed cell migration (Fig. 1e). Besides, western blotting was implemented to examine the impact of FOXM1 on EMT-associated factors, including E-cadherin, N-cadherin, Snail and Slug. As shown in Fig. 1F and Supplementary Fig. S1B, knocking down FOXM1 stimulated E-cadherin level and lessened the levels of N-cadherin, Slug, Snail, ZEB1, and ZEB2. Overexpressing FOXM1 elicited the opposite impacts on above proteins (Supplementary Fig. S1C). Likewise, the enhanced E-cadherin fluorescence intensity and weakened N-cadherin fluorescence intensity under FOXM1 knockdown were obtained through IF staining (Supplementary Fig. S1D).

Moreover, in vivo data revealed that FOXM1 depletion inhibited tumor growth and resulted in lightened tumor weight (Fig. 1g, h). Expressions of Ki-67 and PCNA were lessened in tumors derived from cells under FOXM1 knockdown (Fig. 1i). Moreover, IHC staining indicated that the expression of E-cadherin was enhanced whereas that of N-cadherin was lessened in xenografts with FOXM1 knockdown (Fig. 1i). More importantly, H&E staining assay measured that the number of metastatic nodules was also decreased evidently in mice injected with FOXM1-depleted LUAD cells (Fig. 1j). Meanwhile, the level of E-cadherin protein decreased, while the levels of N-cadherin, Snail, Slug, ZEB1, and ZEB2 proteins increased, in metastatic tumors with FOXM1 knockdown (Fig. 1k and Supplementary Fig. S1E). Thus, we concluded that FOXM1 expression is upregulated in LUAD, and its knockdown prohibited LUAD cell growth and metastasis both in vitro and in vivo.

### FOXM1 induces nuclear translocation of β-catenin and upregulates circ-0039411 in LUAD cells

**Fig. 2:**
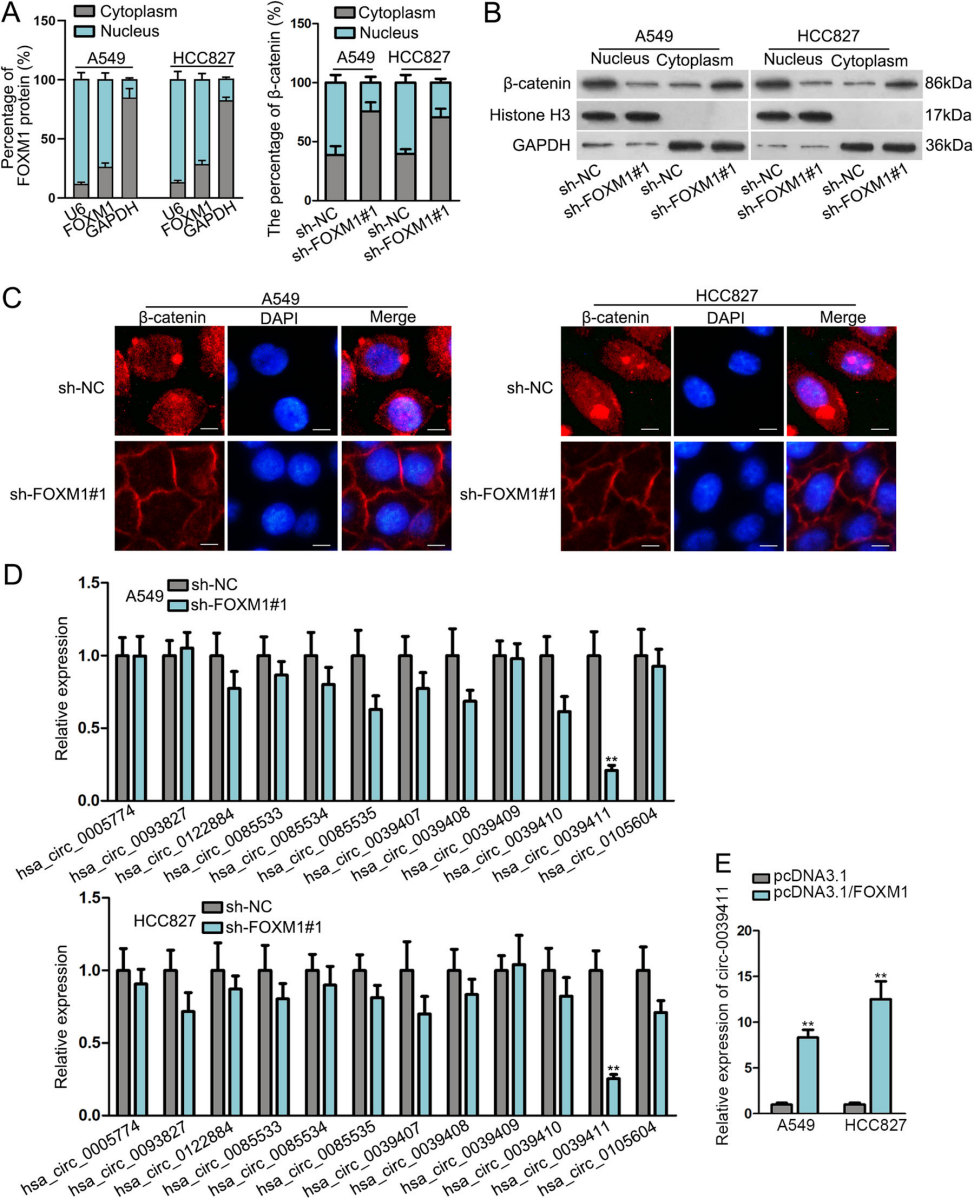
FOXM1 induced β-catenin nuclear translocation and circ-0039411 level in LUAD. **a** Subcellular fractionation analysis was utilized to detect that distribution of FOXM1 protein and β-catenin in LUAD cells. **b, c** Western blot assay and IF assay (scale bar = 30 μm) were utilized to detect the nuclear translocation of β-catenin. GAPDH was the cytoplasmic control and Histone H3 was the nuclear control. **d** qRT-PCR result of circRNA expressions under FOXM1 knockdown. **e** qRT-PCR data of circ-0039411 level under FOXM1 overexpression. **P < 0.01.

Mounting evidences support that FOXM1 contributes to β-catenin nuclear translocation via interacting with β-catenin^23–25^. Entrance of β-catenin into cell nuclei is proved as an essential event for the activation of Wnt pathway, whose activation usually aggravates development and metastasis of various cancers^27,28^. In this regard, we tested the influence of FOXM1 on β-catenin nuclear translocation in LUAD cells. First, subcellular fraction data depicted that FOXM1 protein was mainly distributed in nucleus (while still almost 30% in cytoplasm), and FOXM1 depletion inhibited the level of nuclear β-catenin and caused accumulation of β-catenin in cytoplasm (Fig. 2a). Consistently, western blotting demonstrated that the level of β-catenin in nucleus was decreased, while that in cytoplasm was increased, by silencing FOXM1 (Fig. 2b). Contrarily, overexpressing FOXM1 led to increased nuclear β-catenin and declined cytoplasmic β-catenin (Supplementary Fig. S2A). Moreover, IF images displayed that under FOXM1 deficiency, the concentration of β-catenin in nuclear was lessened and enhanced β-catenin was blocked in cytoplasm (Fig. 2c), which further validated the suppressed nuclear translocation of β-catenin in response to FOXM1 silence. Further, we obtained the enriched β-catenin protein in IP products precipitated by anti-FOXM1, and the harvested FOXM1 protein in IP products induced by anti-β-catenin as well (Supplementary Fig. S2B). In the meantime, IF images presented the co-localization of β-catenin and FOXM1 in both cytoplasm and nucleus of LUAD cells, mainly in nucleus (Supplementary Fig. S2C). Given that we found FOXM1 induced ZEB1 and ZEB2, two pivotal molecules for metastasis in cancers^29,30^, we primarily probed into how FOXM1 modulated their levels in LUAD. Interestingly, we discovered that the luciferase activity of ZEB1 promoter, rather than that of ZEB2 promoter, was repressed under FOXM1 knockdown and enhanced under FOXM1 overexpression (Supplementary Fig. S2D). ZEB1is known as a target of β-catenin/TCF^30,31^, so we assumed that FOXM1 triggered ZEB1 transcription via Wnt/β-catenin pathway, and might regulate ZEB2 via other pathways. Additionally, the effectors downstream of Wnt/β-catenin signaling, including CDK1, SOX2, MMP2 and c-Myc, were decreased by FOXM1 inhibition (Supplementary Fig. S2E). These data jointly verified that FOXM1 bound to β-catenin to help β-catenin translocate into the nuclear of LUAD cells, leading to the activation of Wnt/β-catenin pathway.

To further explain the mechanism downstream of FOXM1, we applied qRT-PCR to detect the expression of circular RNAs (circRNAs) associated with the abovementioned key genes downstream of Wnt/β-catenin pathway, including CDK1 (hsa_circ_0005774, hsa_circ_0093827), SOX2 (hsa_circ_0122884), MYC (hsa_circ_0085533, hsa_circ_0085534, hsa_circ_0085535) and MMP2 (hsa_circ_0039407, hsa_circ_0039408, hsa_circ_0039409, hsa_circ_0039410, hsa_circ_0039411, hsa_circ_0105604). As shown in Fig. 2d, only the level of hsa_circ_0039411 was pronouncedly declined by suppressed FOXM1. Oppositely, FOXM1 overexpression upregulated circ-0039411 level (Fig. 2e). Therefore, we selected circ-0039411 (circ-MMP2) for following researches. Jointly, FOXM1 induces the nuclear translocation of β-catenin and upregulates the expression of downstream circ-0039411 in LUAD.

### Circ-0039411 is transcriptionally activated by FOXM1 in LUAD

**Fig. 3:**
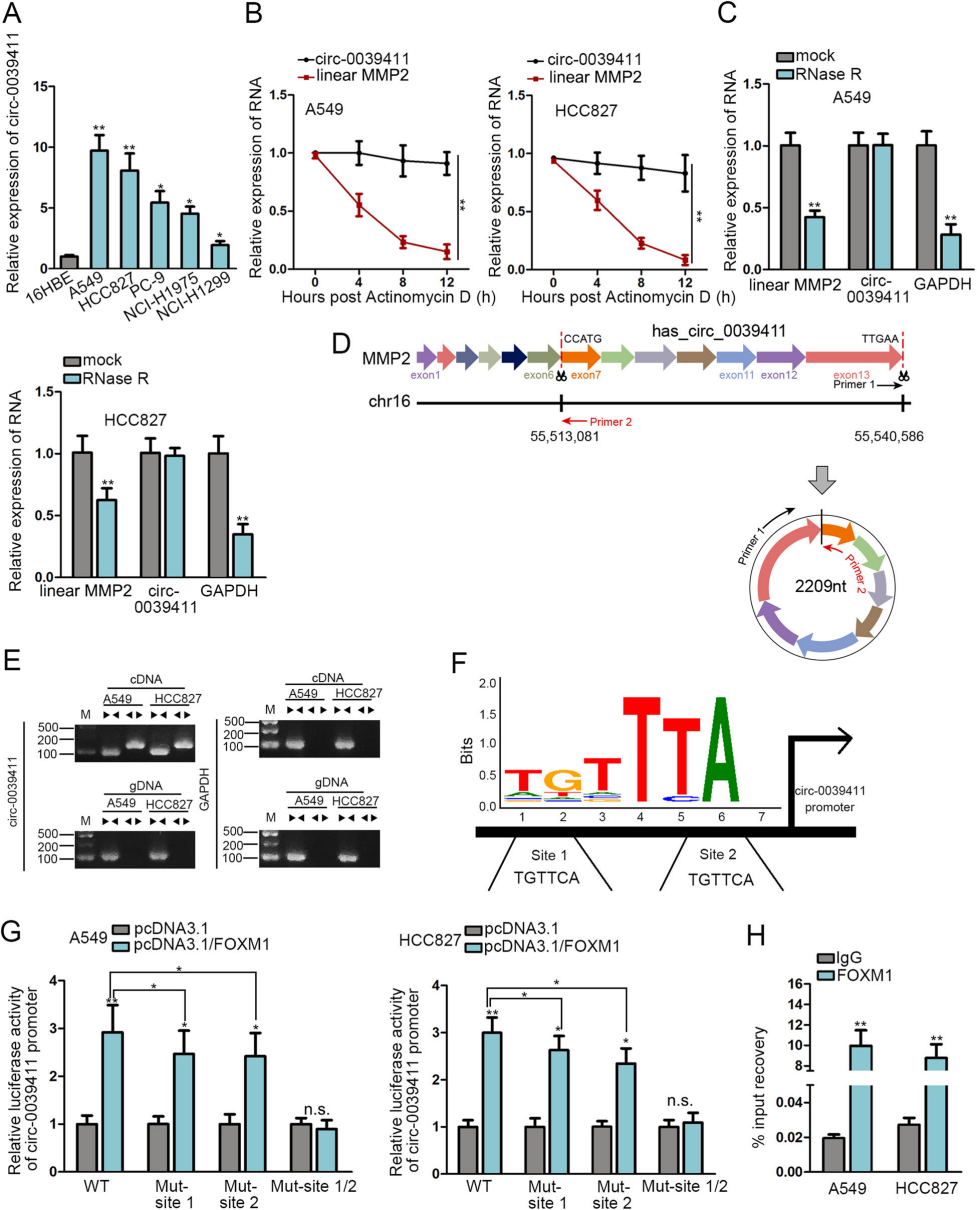
Circ-0039411 transcription was activated by FOXM1 in LUAD. **a** The result of qRT-PCR of circ-0039411 expression in LUAD and normal cells. **b** qRT-PCR was utilized to test RNA expression in LUAD cells after Act D treatment. **c** The qRT-PCR detection of RNA expression after LUAD cells were treated with RNase R. **d** Ring forming diagram of circ-0039411. **e** AGE (agarose gel electrophoresis) results of PCR products amplified by convergent or divergent primers. **f** The binding motif of FOXM1 and the two possible FOXM1 binding sites in circ-0039411 promoter. **g** Luciferase reporter assay was carried out to estimate the impact of FOXM1 on circ-0039411 transcription. **h** qRT-PCR measured the enrichment of circ-0039411 promoter in ChIP products of FOXM1. *P < 0.05, **P < 0.01.

Thereafter, the role of circ-0039411 in LUAD was probed. First, qRT-PCR verified the elevated circ-0039411 expression in LUAD cells relative to normal 16HBE cells (Fig. 3a). Also, circ-0039411 showed high expression trend in LUAD samples versus the para-tumor ones (Supplementary Fig. S3A). More significantly, high circ-0039411 level was linked to unsatisfactory prognosis of LUAD patients (Supplementary Fig. S3B). Moreover, we obtained the positive correlation between circ-0039411 and FOXM1 expressions in LUAD samples (Supplementary Fig. S3C). These observations indicated that circ-0039411 might play a role during LUAD development.

Thereafter, we confirmed the stable characteristic of circ-0039411 as a circRNA. As expected, circ-0039411 expression had no significant change whereas that of linear MMP2 declined remarkably in LUAD cells under treatment with Actinomycin D (Act D) and RNase R (Fig. 3b, c). As presented in Fig. 3d, circ-0039411, with 2209 nt in length, is back spliced from MMP2 gene and covers 7 exons. It was verified that divergent primers generated the circular isoform of circ-0039411 within cDNA rather than genomic DNA (gDNA), while convergent primers amplified the linear isoform in both cDNA and gDNA (Fig. 3e).

Further, we investigated how FOXM1 regulated circ-0039411 level in LUAD. Based on previous findings that FOXM1 functions as a transcription factor in cancers^10,32^, we hypothesized that FOXM1 could affect the transcription of circ-0039411 in LUAD. We obtained promoter sequence of circ-0039411 from UCSC and compared it with FOXM1 binding motif in JASPAR. Consequently, we predicted two possible FOXM1 binding sites in circ-0039411 promoter (Fig. 3f). Then, luciferase reporter assay demonstrated that FOXM1 upregulation strengthened the activity of wild type (WT) circ-0039411 promoter, and mutation of either site 1 or 2 partially rescued above effect, whereas circ-0039411 promoter with the mutation of both site 1 and 2 exhibited no change under FOXM1 overexpression (Fig. 3g). Afterwards, ChIP assay confirmed the binding of FOXM1 to circ-0039411 promoter in both A549 and HCC827 cells (Fig. 3h). In summary, FOXM1 serves as an activator for circ-0039411 transcription in LUAD.

### Circ-0039411 knockdown abrogates proliferation, migration, and EMT in vitro, and prevents tumor growth and metastasis in vivo

**Fig. 4:**
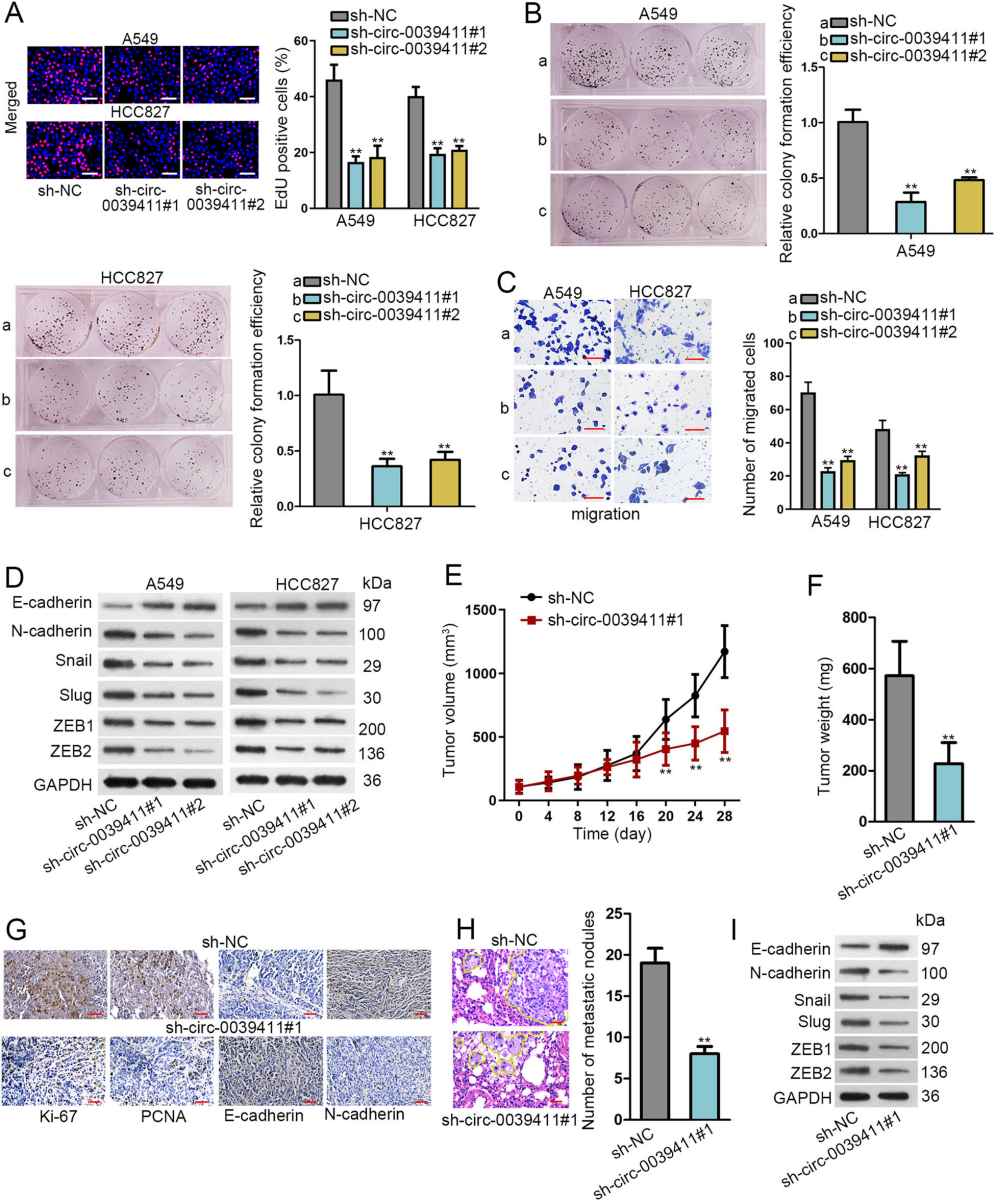
Circ-0039411 knockdown impeded cell proliferation, migration, EMT as well as tumor growth and metastasis in LUAD. **a, b** EdU assay (scale bar = 100 μm) and colony formation experiment were conducted to estimate cell proliferation. **c** Cell migration ability was estimated by transwell assay (scale bar = 100 μm). **d** Western blot assay were carried out for testing EMT. GAPDH was an internal control. **e** Tumor volume in mice was detected after circ-0039411 was knocked down. **f** After circ-0039411 knockdown, tumor volume and weight of xenografts was tested. **g** IHC was carried out to detect proliferation and EMT indexes in xenografts after circ-0039411 knockdown (scale bar = 50 μm). **h** Number of metastatic nodules was detected by H&E staining (scale bar = 50 μm). **i** Western blots of EMT-related proteins in xenografts with circ-0039411 knockdown. **P < 0.01.

We proceeded to test whether altering circ-0039411 level could affect cellular behaviors in LUAD. First, the obvious knockdown of circ-0039411 was obtained in A549 and HCC827 cells after the transfection of sh-circ-0039411#1/#2 (Supplementary Fig. S4A). Then, EdU and colony formation assays measured that circ-0039411 downregulation repressed LUAD cell proliferation (Fig. 4a, b). Moreover, transwell assay illustrated that the migration ability of two LUAD cells was impaired by circ-0039411 suppression (Fig. 4c). Western blot clarified that EMT process were suppressed by circ-0039411 deficiency, supported by decreased levels of N-cadherin, Snail, Slug, ZEB1 and ZEB2 protein and increased level of E-cadherin protein (Fig. 4d and Supplementary Fig. S4B). Also, the fluorescence intensity of E-cadherin increased and that of N-cadherin decreased under circ-0039411 knockdown (Supplementary Fig. S4C). Additionally, downregulating circ-0039411 decreased CDK1, SOX2, MMP2 and c-Myc levels (Supplementary Fig. S4D).

Besides, according to in vivo data in Fig. 4e, f, circ-0039411 downregulation reduced tumor growth. Besides, positivity of Ki-67 and PCNA was diminished in xenografts with circ-0039411 deficiency (Fig. 4g). Meanwhile, the staining of E-cadherin rose whereas that of N-cadherin declined in tumors upon circ-0039411 knockdown (Fig. 4g). Furthermore, H&E staining displayed that the number of metastatic nodules in metastatic tumors obtained from mice injected with circ-0039411-dilenced cells was greatly reduced in comparison to that in control group (Fig. 4h). Additionally, we observed a higher level of E-cadherin protein and lower levels of N-cadherin, Snail, Slug, ZEB1 and ZEB2 in above tumors compared with those from control group (Fig. 4i and Supplementary Fig. S4E). To sum up, circ-0039411 knockdown abrogates LUAD cell proliferation, migration, and EMT in vitro, and prevents LUAD tumor growth and metastasis in vivo.

### Circ-0039411 depends on FOXM1 to affect proliferation, migration, and EMT in LUAD

**Fig. 5:**
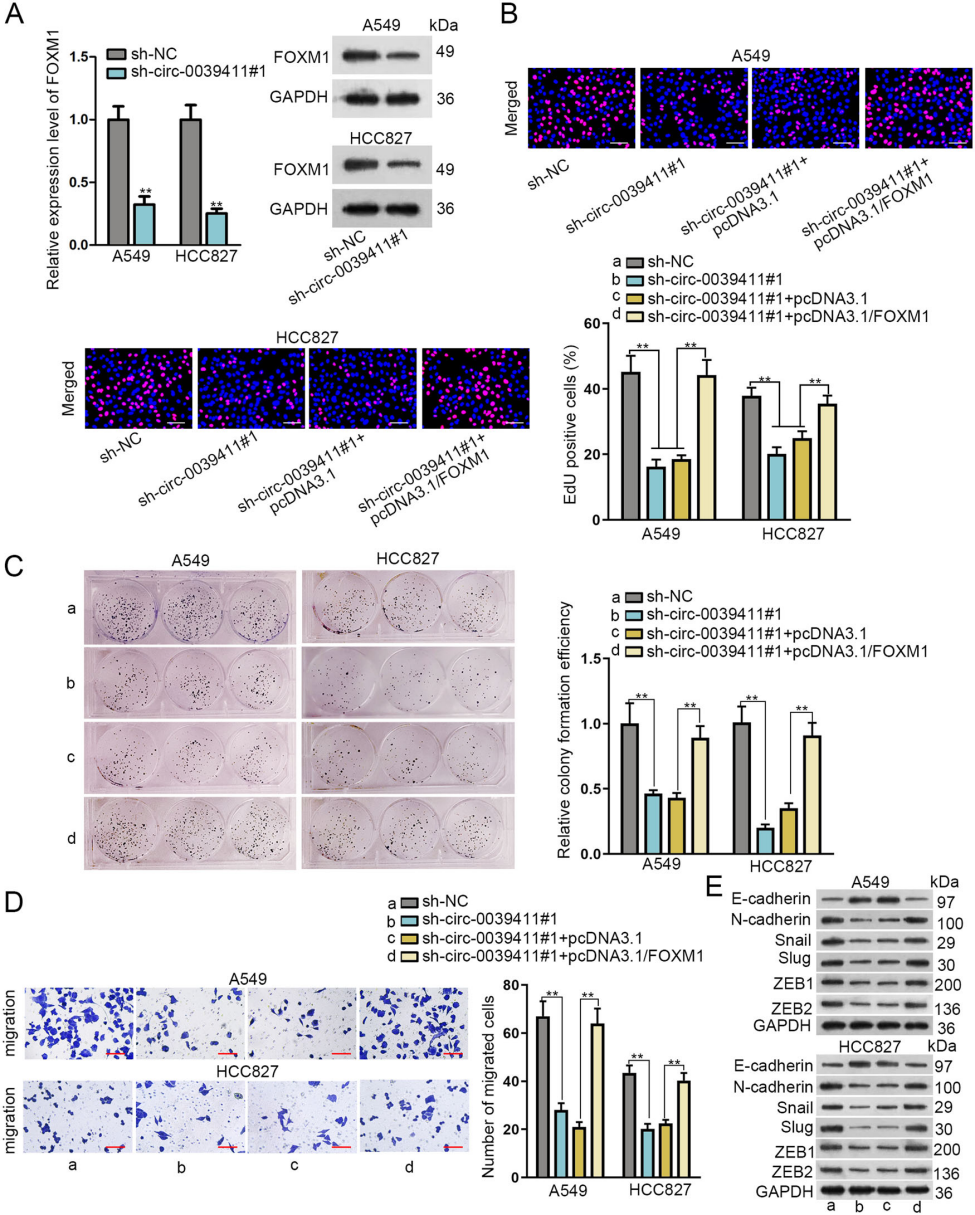
Circ-0039411 affected proliferation, migration, and EMT relying on FOXM1 in LUAD. **a** The qRT-PCR and western blot assays was utilized to detect the effect of circ-0039411 inhibition on FOXM1 expression. GAPDH was the internal control. **b, c** Cell proliferation was detected by EdU (scale bar = 100 μm) and colony formation assays. **d** Transwell assay was utilized to test the cell migration (scale bar = 100 μm). **e** Western blot assay was adopted to detect EMT-related proteins. GAPDH was the internal control. **P < 0.01. n.s. meant no significance.

As widely acknowledged, circRNAs function in cancer development through regulating certain genes. Hence, we tried to test whether circ-0039411 exerted its impacts by targeting FOXM1. First, circ-0039411 knockdown suppressed the mRNA and protein expression of FOXM1 in LUAD cells (Fig. 5a and Supplementary Fig. S5A). Then, several rescue experiments were performed. FOXM1 overexpression fully offset the inhibitory function of circ-0039411 knockdown in LUAD cell proliferation (Fig. 5b, c). FOXM1 upregulation completely reversed the repressing impact of circ-0039411 deficiency on cell migration (Fig. 5d). Besides, co-transfection of pcDNA3.1/FOXM1 fully remedied the changes on EMT-correlated proteins in sh-circ-0039411#1-transfected LUAD cells (Fig. 5e and Supplementary Fig. S5B). Meanwhile, we also verified that the circ-0039411 upregulation could rescue the restraining function of FOXM1 knockdown in cell proliferation, migration and EMT in LUAD (Supplementary Fig. S5C-G). Overall, circ-0039411/FOXM1 axis facilitates cell proliferation, migration, and EMT in LUAD.

### Circ-0039411 enhances the stability of FOXM1 mRNA via recruiting IGF2BP3

**Fig. 6:**
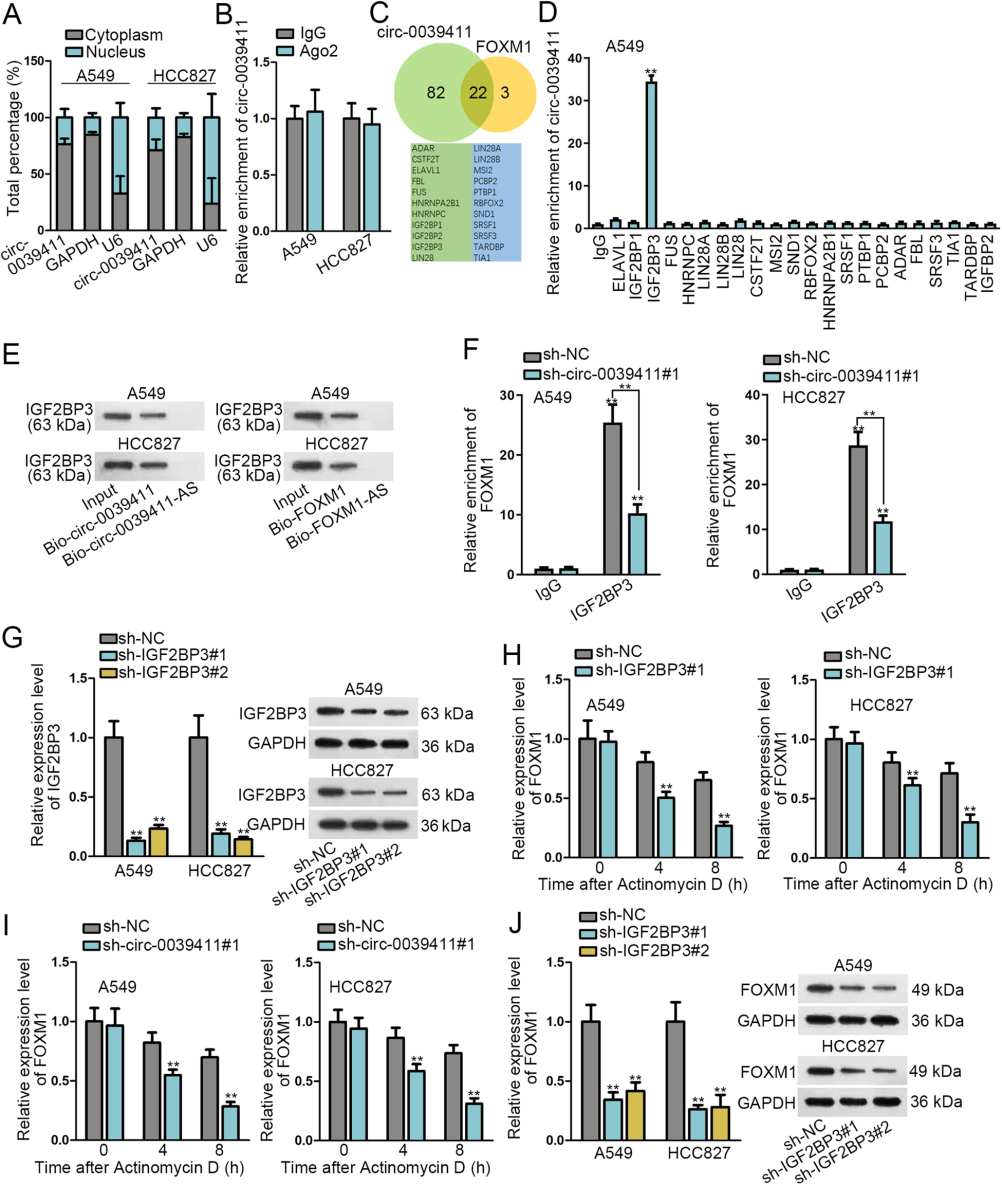
Circ-0039411 enhanced the stability of FOXM1 mRNA via recruiting IGF2BP3. **a** Subcellular fractionation was utilized to test the location of circ-0039411 in A549 and HCC827 cells. **b** RIP assay plus qRT-PCR analysis of circ-0039411 enrichment by anti-Ago2 in A549 and HCC827 cells. **c** StarBase was adopted to screen out RBPs which could combine with circ-0039411 and FOXM1. **d** qRT-PCR of circ-0039411 enrichment precipitated by 22 candidate RBPs via RIP assays. **e** Immunoblot following RNA pull down assay detected the level of IGF2BP3 in the pulldown of different groups. **f** RIP assay revealed the influence of circ-0039411 silence on the binding of IGF2BP3 to FOXM1 in LUAD cells. **g** The qRT-PCR and western blot confirmed the efficiency of silencing IGF2BP3. **h, i** qRT-PCR data of FOXM1 expression at 0, 4, and 8 h after Act D treatment under IGF2BP3 or circ-0039411 silencing. **j** qRT-PCR data and western blots of FOXM1 level under IGF2BP3 knockdown. GAPDH was the internal control. **P < 0.01.

Finally, the mechanism whereby circ-0039411 regulated FOXM1 level was explored. Non-coding RNAs usually affect the level of messenger RNA (mRNA) through cooperating with certain RNA binding protein (RBP)^33^ or via competing endogenous RNA (ceRNA) network^22^. First of all, subcellular fraction assay measured that circ-0039411 mainly distributed in cytoplasm (Fig. 6a). Further, RIP assay detected that precipitates of anti-Ago2 group recovered few enrichment of circ-0039411 (Fig. 6b), excluding the potential for circ-0039411 as a ceRNA in LUAD. Hence, we aimed to find out the probable cooperating RBP for circ-0039411. Through utilizing starBase, 22 common RBP that possibly bound with both circ-0039411 and FOXM1 were screened out (Fig. 6c). However, RIP assay displayed that circ-0039411 was only obviously enriched in anti-IGF2BP3 group (Fig. 6d). Therefore, IGF2BP3 was selected as the focus of following assays. After that, we validated that IGF2BP3 was enriched in the complexes pulled down by Bio-circ-0039411 (sense) and Bio-FOXM1 (sense), instead of in the compounds by Bio-circ-0039411-AS (anti-sense) or Bio-FOXM1-AS (anti-sense) (Fig. 6e). Besides, the enrichment of FOXM1 precipitated by IGF2BP3 was decreased when knocking down circ-0039411 (Fig. 6f). In addition, the efficient knockdown of IGF2BP3 was testified by qRT-PCR and western blot (Fig. 6g and Supplementary Fig. S6A). Following Act D treatment, knocking down either IGF2BP3 or circ-0039411 expedited the degradation of FOXM1 mRNA (Fig. 6h, i). Also, the mRNA and protein level of FOXM1 was attenuated by IGF2BP3 depletion (Fig. 6j and Supplementary Fig. S6B). As a whole, circ-0039411 enhances the stability of FOXM1 via recruiting IGF2BP3 in LUAD, indicating circ-0039411-FOXM1 formed positive feedback loop.

## Discussion

Though surgery, chemotherapy and radiotherapy have been improved during last decades, the survival rate of LUAD remains low^34,35^. As a result, it is imperative for us to research molecular mechanisms related to LUAD progression and develop novel methods for the treatments of LUAD patients.

Former works have established the relation between FOXM1 and LUAD development by illustrating its impact on cell growth, chemo-resistance, and metastasis^13–15^. Also, poor prognosis in LUAD is proved be to linked to high FOXM1 level^36^. These findings indicated the pivotal role of FOXM1 in LUAD, and meant that the mechanism of FOXM1 in LUAD deserves further explanation. Consistently, the current study confirmed FOXM1 upregulation in LUAD based on TCGA data and demonstrated high FOXM1 level in LUAD cells, validating its link to LUAD. In vitro experiments presented the suppressive function of FOXM1 knockdown on proliferation, migration and EMT, and in vivo data supported that FOXM1 silence slowed down tumorigenesis and metastasis in LUAD. These results confirmed the carcinogenic role of FOXM1 in LUAD.

Referring to previous studies, FOXM1 binds to β-catenin and facilitates its nuclear translocation in tumor cells, such as in leukemia, osteosarcoma, glioma, and lung cancer^23–25,37^. Nuclear import of β-catenin is axiomatically known to be essential in the Wnt/β-catenin pathway-mediated cancer development^27,28^. Our data concordantly affirmed that FOXM1 contributed to the nuclear transport of β-catenin in LUAD through its direct binding to β-catenin. Moreover, we validated the activation of Wnt/β-catenin under FOXM1 overexpression by observing the elevated levels of several downstream molecules including CKD1, SOX2, MMP2, and c-Myc. Also, the EMT-related molecules ZEB1 and ZEB2 were upregulated by FOXM1, which was consistent to previous findings^38–40^. However, we discovered that only ZEB1 was activated by FOXM1 at transcription level. Formerly, there is also a former study confirmed that FOXM1 cannot affect ZEB2 promoter transcription in lung cancer cells^41^. Considering the known knowledge that ZEB1 is a target of β-catenin/TCF^30,31^, we suggested that FOXM1 regulated ZEB1 transcription via Wnt/β-catenin signaling. As to the confirmed effect of FOXM1 on the protein level of both ZEB1 and ZEB2, we assumed that FOXM1 might have other ways to regulate ZEB1 and ZEB2, such as through miR-200b as proved by former findings^42^.

Circular RNAs (circRNAs) are promising potential biomarkers because of their unique structure, high stability, and specific expression patterns^43^. It has been reported that circRNAs are aberrantly expressed in tumors and function as tumor suppressors or oncogenes in diverse cancers^44–46^, including LUAD^47^. In addition, transcription factor exerts important function in regulating the transcription of molecules including circRNAs, so as to affect diverse disease including cancer. For instance, circ-4099 was transcriptionally upregulated by TNF-alpha-induced GRP78 in intervertebral disc degradation^48^. ERα induced circ_0023642 level in bladder cancer at transcription level^49^. c-Fos transcriptionally upregulated circPVT1 in non-small cell lung cancer^50^. Herein, we first tested the impact of FOXM1 on circRNAs associated with the downstream genes of Wnt/β-catenin pathway, finding that circ-0039411 was transcriptionally activated by FOXM1 in LUAD. Notably, we confirmed the high circ-0039411 level in LUAD samples and cells and revealed its prognostic value through Kaplan-Meier analysis. These data provided a novel circRNA that potentially served as a prognostic marker in LUAD. Functionally, we delineated that circ-0039411 knockdown abrogated LUAD cell proliferation, migration and EMT in vitro and impaired tumorigenesis and metastasis in vivo. Data from rescue assays suggested that circ-0039411 exerted its function in LUAD relying on FOXM1.

RBPs usually cooperates with ncRNAs to enhance the stability of mRNAs^33^. In this research, we first confirmed that circ-0039411 mainly distributed in cytoplasm via subcellular fraction assay and also excluded the ceRNA regulation pattern of circ-0039411 via Ago2-RIP assay. Then with utilization of starBase, we obtained several common RBPs of circ-0039411 and FOXM1, and then IGF2BP3 was screened out. We further verified IGF2BP3 that bound to both circ-0039411 and FOXM1 in LUAD cells. Formerly, the role of IGF2BP3 as a contributor of mRNA stability has been widely reported^51,52^. However, the association of IGF2BP3 with FOXM1 and circ-0039411 was not uncovered until this work. We discovered that circ-0039411 bound to IGF2BP3 and facilitated its mRNA-stabilizing function on FOXM1.

**Fig. 7:**
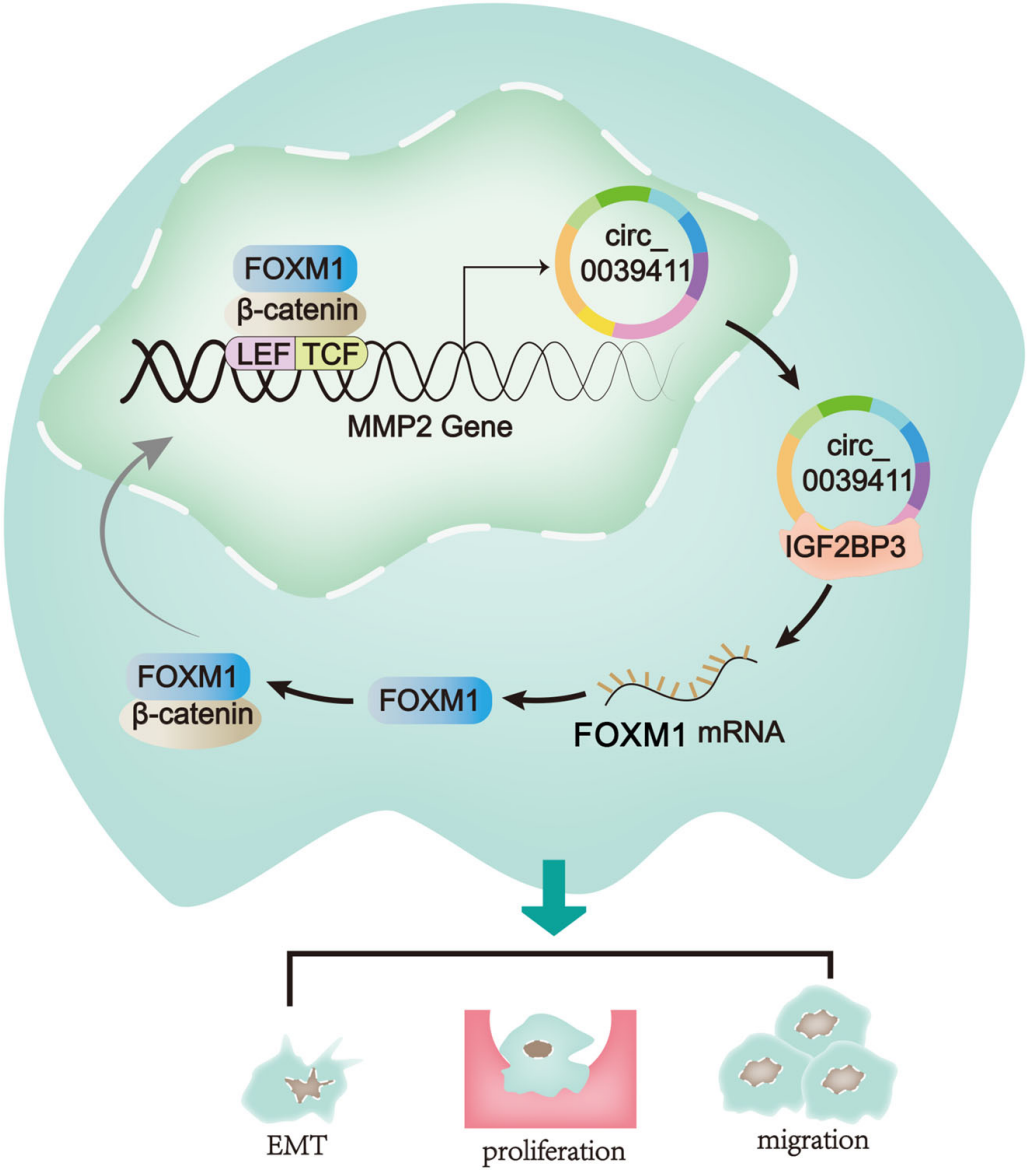
Graphical description of the FOXM1/circ-0039411/IGF2BP3/FOXM1 loop in LUAD cells. FOXM1 promotes the nuclear translocation of β-catenin as well as the transcription of a circRNA (circ-0039411) originated from MMP2, a downstream target of Wnt pathway. Circ-0039411 upregulation facilitates LUAD cell proliferation, migration and EMT. Besides, circ-0039411 enhances the stability of FOXM1 mRNA via recruiting IGF2BP3, thus forming a positive feedback loop.

In a conclusion, this study provided novel data to show that FOXM1 promoted nuclear translocation of β-catenin as well as the transcription of a circRNA (circ-0039411) from MMP2, a downstream target gene of Wnt pathway. Circ-0039411 upregulation facilitated LUAD cell proliferation, migration and EMT. Besides, circ-0039411 enhances the stability of FOXM1 via recruiting IGF2BP3, thus forming a positive feedback loop in regulating LUAD progression (Fig. 7). In a word, circ-MMP2 (circ-0039411) induced by FOXM1 promotes the proliferation and migration of LUAD cells in vitro and in vivo, which might provide some novel thoughts for molecular mechanism researches regarding LUAD. However, the limits of this study are that the direct function of circ-0039411 on LUAD is still needed to be validated under a FOXM1 knockout condition. The mechanism of FXOM1 regulating ZEB1 and ZEB2 in LUAD is required to be explained in detail. To better validate the value of circ-0039411/FOXM1 axis in LUAD, we will dedicate ourselves to address points in the near future.

## Materials and methods

### Patient samples

LUAD samples and matched para-tumor ones were obtained from 74 LUAD patients in Linyi People’s Hospital, and patients had all signed informed consents. The patients underwent no radio- or chemo-therapy before taking the surgery. The experiment was permitted by the ethics committee of Linyi People’s Hospital. Samples after dissection were maintained under −80 °C after immediate frozen by liquid nitrogen.

### Cell culture and treatment

Five human LUAD cell lines (A549, HCC827, PC-9, NCI-H1975, and NCI-H1299) and human bronchial epithelial cell line (16HBE) were procured from the American Type Cell Culture (ATCC, Rockville, Maryland) and cultured at 37 °C in 5% CO_2_. Cell samples were all propagated in DMEM medium (Gibco, Grand Island, NY) adding with10% fetal bovine serum (FBS, Gibco). For treating A549 and HCC827 cells, 10 mg/mL of Actinomycin D (Act D) was bought from Sigma-Aldrich (St. Louis, MO), and 3 U/μg of RNase R was acquired from Epicentre Technologies (Madison, WI).

### RNA extraction and quantitative real-time polymerase chain reaction (qRT-PCR)

A549 and HCC827 cell samples were suspended in 1 mL of TRIzol Reagent (Invitrogen, Carlsbad, CA), and then total RNAs were reverse-transcribed to cDNA as per the instruction. Gene expression levels were measured by qRT-PCR with SYBR Green Taq Mix (Takara, Shiga, Japan) using 2^–∆∆Ct^ method. GAPDH or U6 was used as the internal control. The primer sequences and reference sequences for primer design are presented in Supplementary file 1.

### Cell transfection

The specific shRNAs (short hairpin RNAs) and control shRNAs were designed and produced by Genepharma Company (Shanghai, China), applying for silencing FOXM1, circ-0039411 and IGF2BP3 in inidcated cells. Besides, the pcDNA3.1 vector and pcDNA3.1 (+) CircRNA Mini Vector were acquired from Genepharma to overexpress FOXM1 and circ-0039411, respectively. Transfection was conducted for 48 h using Lipofectamine2000 (Invitrogen).

### 5-Ethynyl-2′-deoxyuridine (EdU) assay

Transfected cell samples were placed on the sterile coverslips in 24-well plates and then processed with the EdU assay kit (Ribobio, Guangzhou, China) as per the guidebook. After nuclear counterstaining with DAPI, images were taken with fluorescence microscopy (Olympus, Tokyo, Japan).

### Colony formation assay

Clonogenic cells in 6-well plates were plated at 500 cells per well for 14 days of culturing. Then clones were fixed in 4% paraformaldehyde and stained in 0.1% crystal violet for manual counting.

### Transwell migration assay

Cell samples cultured in serum-free medium were placed to the upper chamber of transwell insert (Millipore, Bedford, MA), and lower chamber was supplemented with complete medium containing 10% FBS. After incubated for 24 h, cells migrated to the lower chamber were fixed and stained in 0.1% crystal violet for imaging under microscopy.

### Western blot

Cells were lysed in RIPA (radioimmunoprecipitation assay) lysis buffer, separated on 12% SDS-PAGE (sodium dodecyl sulfate polyacrylamide gel electrophoresis) and shifted to PVDF (Polyvinylidene Fluoride) membranes (Millipore). After blocked in 5% skim milk, membranes were probed with the primary antibodies against loading control GAPDH (1:10,000, ab818602), Slug (1:2000, ab106077), Snail (1:1000, ab216347), N-cadherin (1:500, ab98952), E-cadherin (1:10,000, ab40772), β-catenin (1:5000, ab32572), Histone H3 (1:1000, ab1791), FOXM1 (1:1000, ab207298), and IGF2BP3 (1:1000, ab177477), followed by further incubation with corresponding secondary antibodies conjugated to HRP (all from Abcam, Cambridge, MA). Protein signals were monitored by ECL Substrate (Pierce, Rockford, IL).

### Immunofluorescence (IF) assay

Cells were prepared on the culture slide for IF assay, rinsed in PBS and fixed. Following blocked in 5% BSA, cell samples were probed severally with the primary antibodies targeting E-cadherin (1:100, ab194982, Abcam) and N-cadherin (1: 200, ab98952, Abcam), β-catenin (1:100, #8480, Cell Signaling Technology) and FOXM1 (1:250, ab207298, Abcam) first and with corresponding secondary antibodies next. After washing, slides were processed with DAPI and analyzed under microscopy.

### Subcellular fractionation assay

The separation of nucleus-cytoplasm in A549 and HCC827 cells was achieved as per the manual of PARIS Kit (Invitrogen). For quantification of RNAs in indicated fractions, GADPH and U6 were employed as the fractionation indicators.

### Luciferase reporter assay

The circ-0039411 promoter wild-type with potential FOXM1 binding sites or mutated sites were acquired and fused with pGL3 luciferase reporter vectors (Promega, Madison, WI). The promoter reporter of ZEB1 and ZEB2 were established by inserting their promoter sequences into pGL3 luciferase reporter vectors, respectively. A549 and HCC827 cells in 96-well plates were co-transfected with luciferase vectors and pcDNA3.1/FOXM1 or NC pcDNA3.1 for 48 h, followed by activity analysis via Dual-Luciferase Reporter Assay System (Promega).

### Chromatin immunoprecipitation (ChIP) assay

As instructed by supplier, EZ-CHIP KIT (Millipore) was acquired for RIP assay using anti-FOXM1 (1:100, #20459, Cell Signaling Technology) antibody or anti-IgG antibody (#3900, Cell signaling Technology). qRT-PCR was performed for quantification of precipitated chromatin collected by adding beads.

### RNA immunoprecipitation (RIP) assay

Cell lysates from RIP lysis buffer were prepared for the incubation in RIP buffer with the beads conjugated with antibodies including anti-Ago2, anti-IGF2BP3 or anti-IgG. Anti-IgG antibody served as the control. Followed by protein digestion, purified RNAs were examined by qRT-PCR.

### Co-immunoprecipitation (CoIP)

Protein Co-IP assays were performed as described previously. In short, lysates from cultured A549 and HCC827 cells were obtained using RIPA and underwent immunoprecipitation with anti-FOXM1 (1:50, #20459, Cell signaling Technology), anti-β-catenin (1:50, #8480, Cell signaling Technology) or anti-IgG (1:20, #3420, Cell signaling Technology) for 1 h at 37 °C, and protein A-agarose was added for overnight incubation of lysates. Later, complex of protein A-agarose-antigen-antibody was collected using centrifugation for 2 min and underwent wash immunoprecipitation-HAT buffer for 5 times. The binding proteins were tested by western blot.

### RNA pull down assay

In vitro biotin-labeled RNAs (circ-0039411 and circ-0039411 antisense, FOXM1 and FOXM1 antisense) were transcribed and purified for incubation with cellular protein extracts. The pull-down protein was determined via western blot.

### Animal study

Animal study was approved by the Committee on the Use of Live Animals of Linyi People’s Hospital (reference number: AN-IACUC-2019-023). Six-week-old male BALB/c-nu mice (5 in each group; Vital River, Beijing, China) were housed under SPF-condition for subcutaneous injection with transfected LUAD cells for 28 days. Tumor volume was recorded every 4 days and tumor weight was acquired after killing mice. With respect to in vivo tumor metastasis experiment, mice were injected with transfected LUAD cells via tail vein. Six weeks later, lungs were excised from above mice and kept in formalin for analysis. Number of metastatic nodules in lung was observed under microscope via hematoxylin and eosin (H&E) staining.

### Immunohistochemistry (IHC) assay

The tumor tissue samples from animal study were fixed by 4% paraformaldehyde for embedding in paraffin. Then, the consecutive 4-μm paraffin-embedded sections were fixed and processed with antibodies against Ki-67 (1:500, ab92742) and PCNA (1:100, ab92552), N-cadherin (1:200, ab98952), and E-cadherin (1:500, ab40772) from Abcam, followed by incubation with corresponding secondary antibodies for 30 min at 37 °C.

### Statistical analyses

Correlation between patient survival and gene expression was evaluated by Kaplan-Meier analysis and log-rank test. Expression correlation was tested by Pearson’s correlation analysis. Bio-triplications were conducted for all assays, with data expressed as mean ± standard deviation (SD). Group difference was processed with t-test or one-way ANOVA via applying Graphpad Prism 6 software, with *P* < 0.05 as statistically significant.

## Supplementary information

Figure S1

Figure S2

Figure S3

Figure S4

Figure S5

Figure S6

Supplementary Figure Legends

Supplementary file 1
