# Supplementary figures and images for: Circ-MMP2 (circ-0039411) induced by FOXM1 promotes the proliferation and migration of lung adenocarcinoma cells in vitro and in vivo
Source: Cell Death Dis. 2020 Jun 8;11(6):426. doi: 10.1038/s41419-020-2628-4 (PMC7280516; doi:10.1038/s41419-020-2628-4)

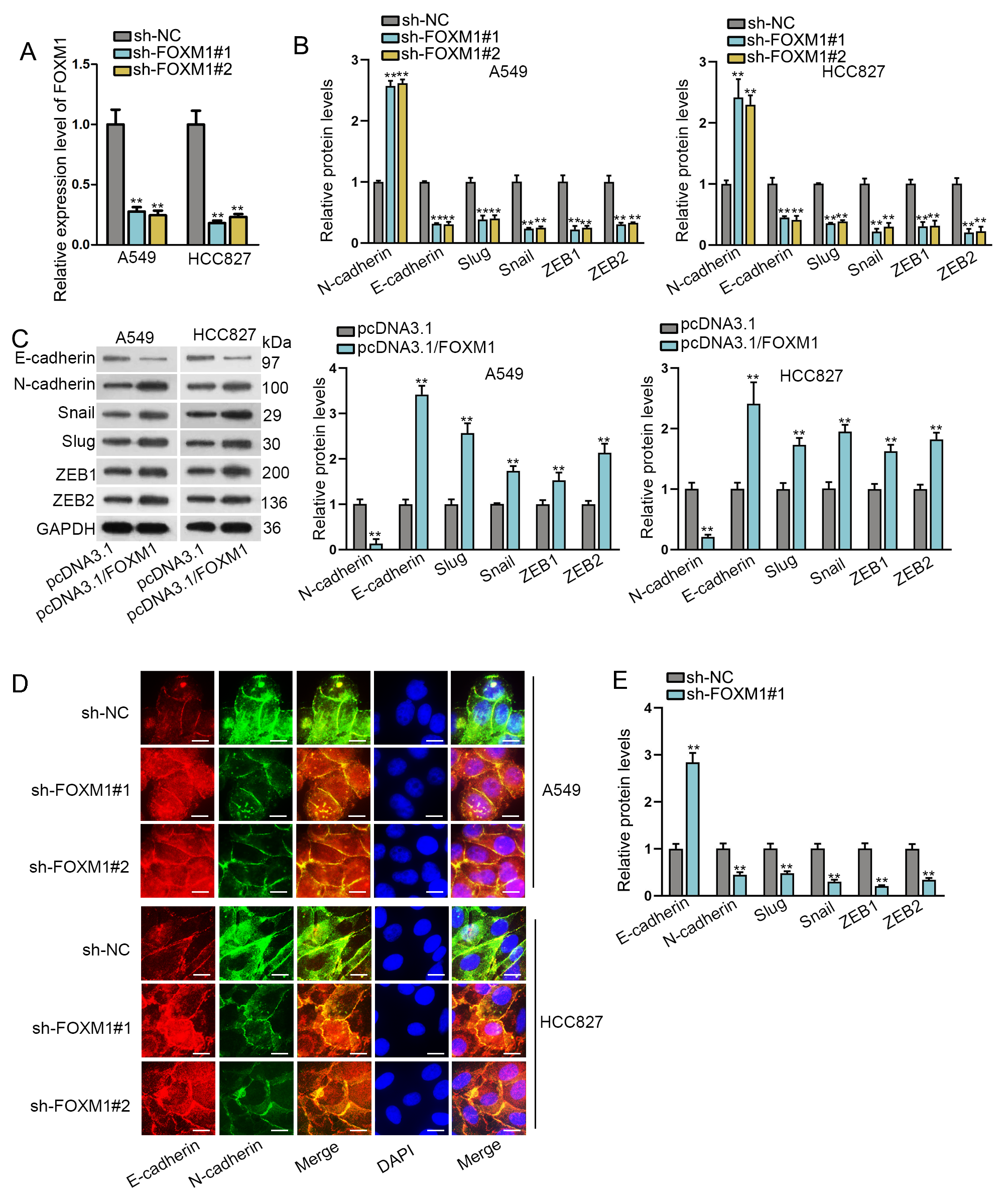

Supplement: Supplementary file 1 — Figure S1 [file 41419_2020_2628_MOESM1_ESM.tif]

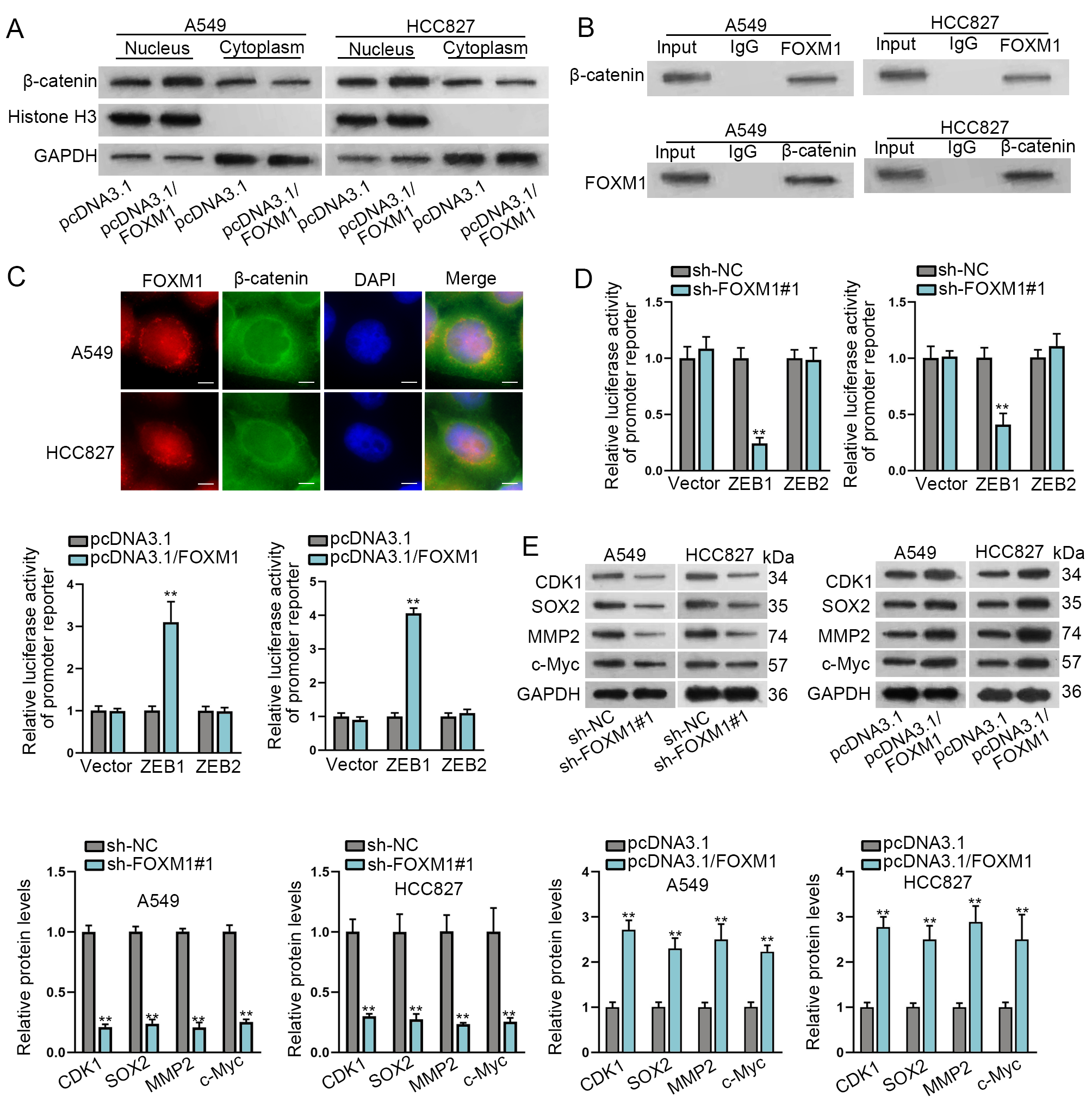

Supplement: Supplementary file 2 — Figure S2 [file 41419_2020_2628_MOESM2_ESM.tif]

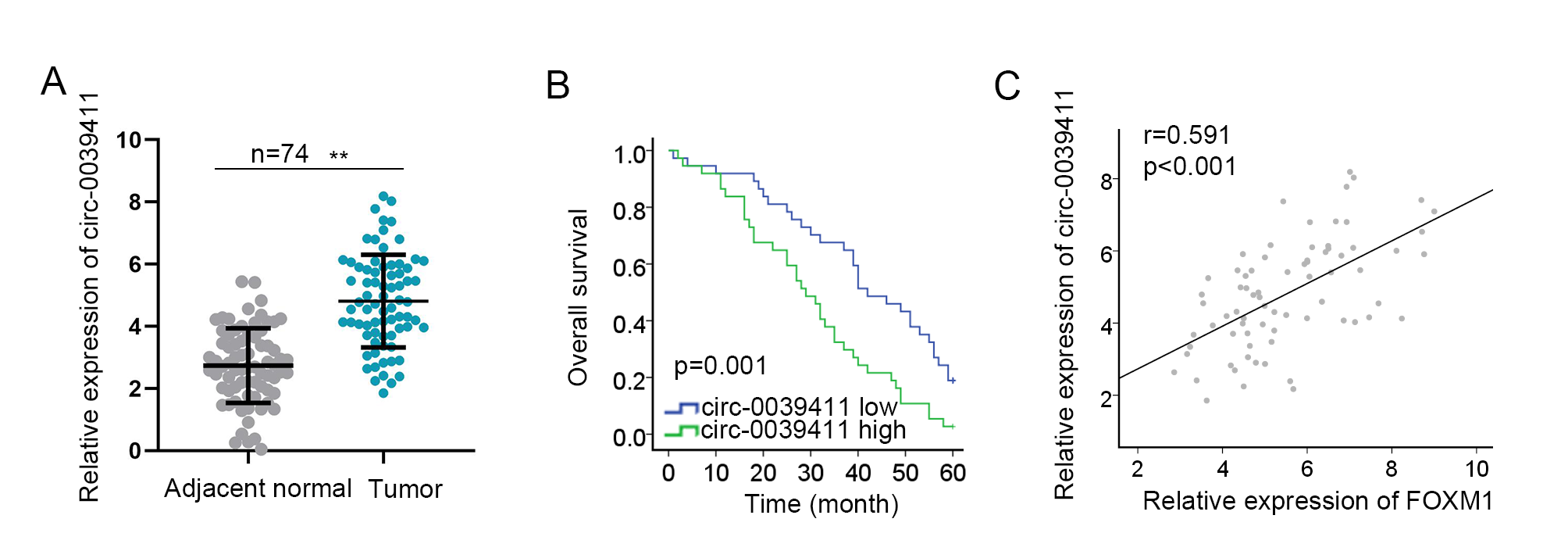

Supplement: Supplementary file 3 — Figure S3 [file 41419_2020_2628_MOESM3_ESM.tif]

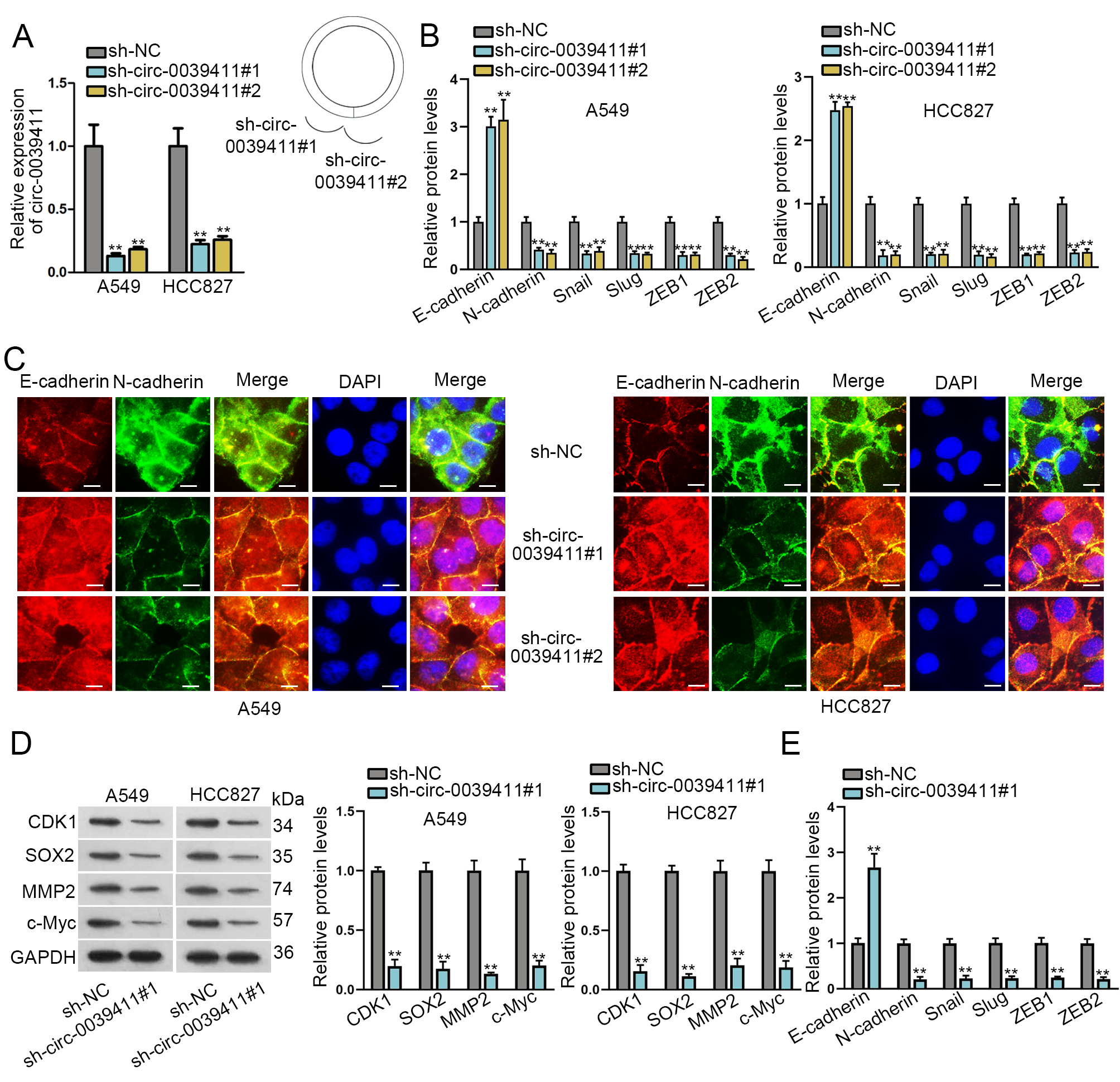

Supplement: Supplementary file 4 — Figure S4 [file 41419_2020_2628_MOESM4_ESM.tif]

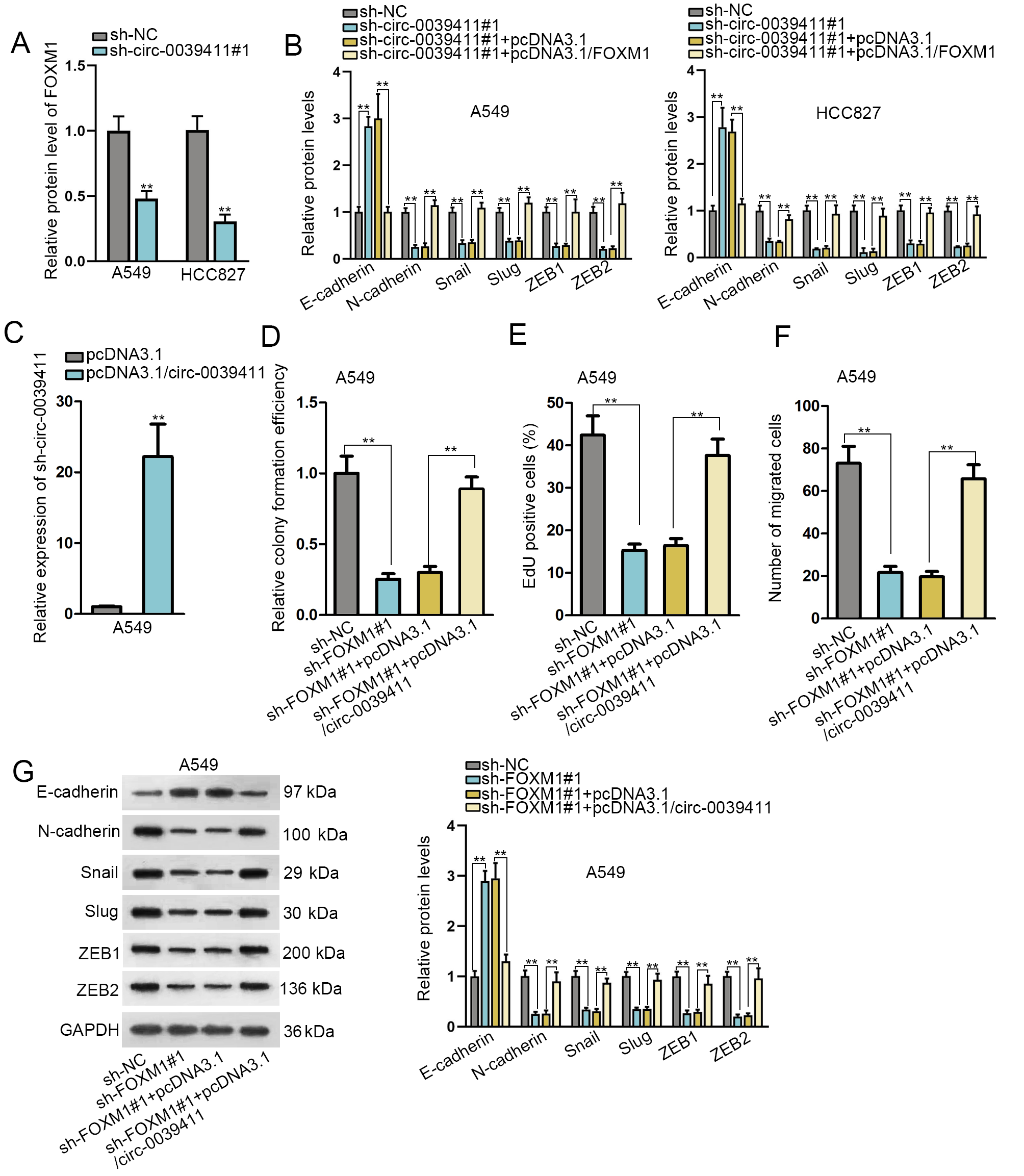

Supplement: Supplementary file 5 — Figure S5 [file 41419_2020_2628_MOESM5_ESM.tif]

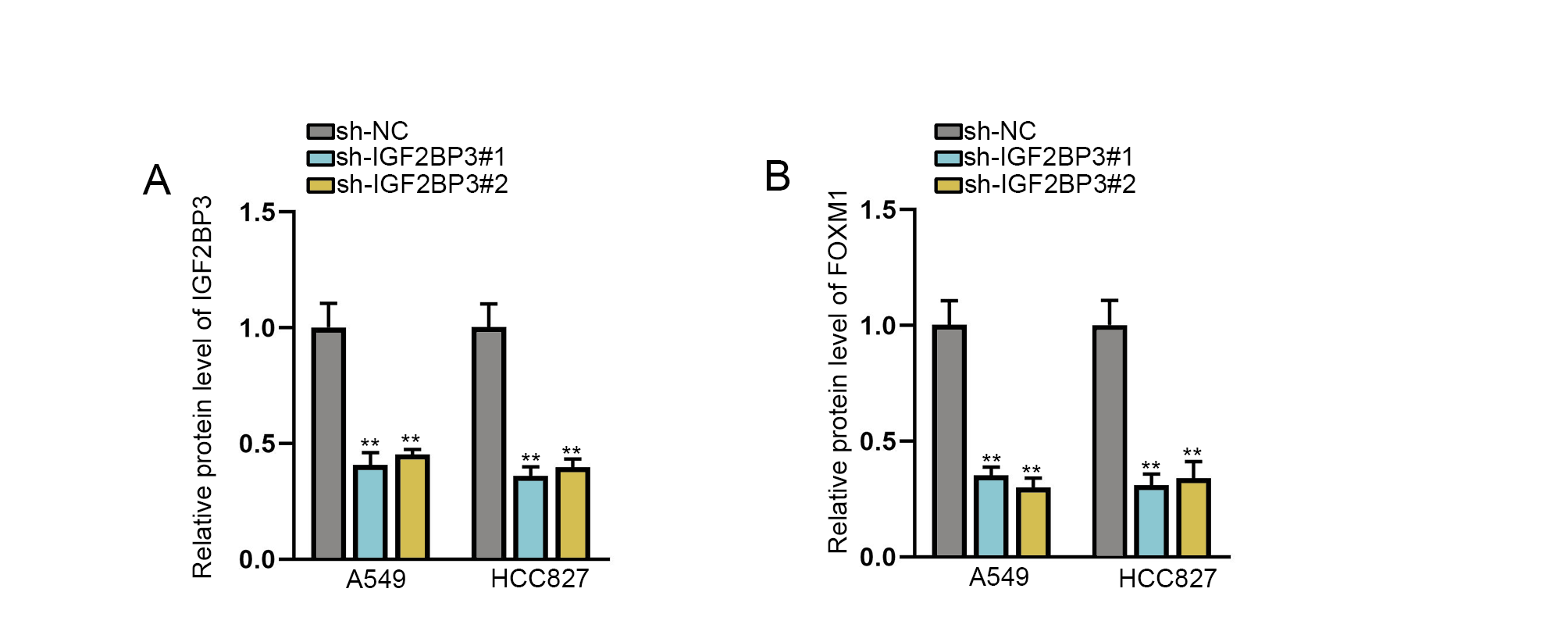

Supplement: Supplementary file 6 — Figure S6 [file 41419_2020_2628_MOESM6_ESM.tif]
