# Supplementary material for: Circ-MMP2 (circ-0039411) induced by FOXM1 promotes the proliferation and migration of lung adenocarcinoma cells in vitro and in vivo
Source: Cell Death Dis. 2020 Jun 8;11(6):426. doi: 10.1038/s41419-020-2628-4 (PMC7280516; doi:10.1038/s41419-020-2628-4)
Supplement: Supplementary file 7 — Supplementary Figure Legends [file 41419_2020_2628_MOESM7_ESM.docx]

**Figure S1**

(A) The transfection efficiency of silencing FOXM1 was tested through qRT-PCR. (B) Quantification of western blot in Figure 1F. (C) Western blots and quantification of EMT-related proteins in LUAD cells under FOXM1 overexpression. (D) IF analysis of E-cadherin and N-cadherin level in LUAD cells under FOXM1 inhibition (scale bar = 30 μm). (E) Quantification of western blots in Figure 1K. ^**^P < 0.01.

**Figure S2**

^­^(A) Western blots of β-catenin in nucleus and cytoplasm of LUAD cells under FOXM1 overexpression, GAPDH and Histone H3 were respective cytoplasmic and nuclear references. (B) Immunoblot of β-catenin in the IP products of FOXM1 and immunoblot of FOXM1 in the IP products of β-catenin in LUAD cells. (C) IF images of co-localization of FOXM1 and β-catenin in LUAD cells (scale bar = 20 μm). (D) Luciferase activity of ZEB1 and ZEB2 promoter reporter under FOXM1 knockdown or overexpression. (E) Western blots and quantification of effectors downstream of Wnt/β-catenin under FOXM1 knockdown and overexpression. ^**^P < 0.01.

**Figure S3**

(A) qRT-PCR of circ-0039411 level in clinical LUAD samples versus adjacent normal ones. (B) Kaplan-Meier curve revealed the relation between high circ-0039411 level and dismal survival of LUAD patients. (C) Pearson’s correlation curve showed the positive association between FOXM1 and circ-0039411 in LUAD samples. ^**^P < 0.01.

**Figure S4**

(A) The circ-0039411 knockdown efficiency was tested by qRT-PCR. (B) Western blot quantification for Figure 4D. (C) IF images of E-cadherin and N-cadherin under circ-0039411 knockdown (scale bar = 30 μm). (D) Western blot images and quantification of CDK1, SOX2, MMP2, and c-Myc proteins under circ-0039411 knockdown. (E) Western blot quantification for Figure 4I. ^**^P < 0.01.

**Figure S5**

(A) Western blot quantification for Figure 5A (B) Western blot quantification for Figure 5E. (C) qRT-PCR was conducted to test the circ-0039411 overexpression efficiency. (D-E) Cell proliferation under different conditions was evaluated via colony formation and EdU assays. (F) Quantification of migrated cells under indicated transfections obtained from transwell assay. (G) Western blot and quantification of EMT markers. GAPDH was the internal control. ^**^P < 0.01. n.s. meant no significance.

**Figure S6**

(A-B) Western blot quantification for Figure 6G and J. ^**^P < 0.01.
