# Supplementary material for: Circ-MMP2 (circ-0039411) induced by FOXM1 promotes the proliferation and migration of lung adenocarcinoma cells in vitro and in vivo
Source: Cell Death Dis. 2020 Jun 8;11(6):426. doi: 10.1038/s41419-020-2628-4 (PMC7280516; doi:10.1038/s41419-020-2628-4)
Supplement: Supplementary file 8 — Supplementary file 1 [file 41419_2020_2628_MOESM8_ESM.docx]

**hsa_circ_0005774**

Divergent primer: TTGGATTCTATCCCTCCTGGT (left)

CAACTCCATAGGTACCTTCTCCA (right)

Junction sequence (3' End - 5' End of circRNA)

>hsa_circ_0005774

TTCCAGGTTATATCTCATCTTTGAGTTTCTTTCCATGGATCTGAAGAAATACTTGGATTCTATCCCTCCTGGTCAGTACATGGATTCTTCACTTGTTAAGGATCTACCATACCCATTGACTAACTATGGAAGATTATACCAAAATAGAGAAAATTGGAGAAGGTACCTATGGAGTTGTGTATAAGGGTAGACACAAAACT

**hsa_circ_0093827**

Divergent primer: AAATGGAAACCAGGAAGCCTA (left)

TCCTGTAAAGATTCCACTTCTGG (right)

Junction sequence (3' End - 5' End of circRNA)

>hsa_circ_0093827

CCAAATGGAAACCAGGAAGCCTAGCATCCCATGTCAAAAACTTGGATGAAAATGGCTTGGATTTGCTCTCGAGCTTTGGGCACTCCCAATAATGAAGTGTGGCCAGAAGTGGAATCTTTACAGGACTATAAGAATACATTTC

**hsa_circ_0122884**

Divergent primer: GTCATTTGCTGTGGGTGATG (left)

CGATGCCGACAAGAAAACTT (right)

Junction sequence (3' End - 5' End of circRNA)

>hsa_circ_0122884

TGTGGATGGGATTGGTGTTCTCTTTTGCAGCTGTCATTTGCTGTGGGTGATGGGATTTTTTTTTTTCCTTTTTCTTTTTGAGCGTACCGGGTTTTCTCTGTACAAAAATAGTCCCCCAAAAAGAAGTCCAGGATCTCTCTCATAAAAGTTTTCTTGTCGGCATCGCGGTTTTTGCGTGAG

**hsa_circ_0085533**

Divergent primer: GCTGCTTAGACGCTGGATTT (left)

AGAAGCCCTGCCCTTCTC (right)

Junction sequence (3' End - 5' End of circRNA)

>hsa_circ_0085533

TTTGGGGACACTTCCCCGCCGCTGCCAGGACCCGCTTCTCTGAAAGGCTCTCCTTGCAGCTGCTTAGACGCTGGATTTTTTTCGGGTAGTGGAAAACCAGGACCCCCGAGCTGTGCTGCTCGCGGCCGCCACCGCCGGGCCCCGGCCGTCCCTGGCTCCCCTCCTGCCTCGAGAAGGGCAGGGCTTCTCAGAGGCTTGGC

**hsa_circ_0085534**

Divergent primer: AAAATAACTGGCAAATATATCATTGAG (left)

AGAAGCCCTGCCCTTCTC (right)

Junction sequence (3' End - 5' End of circRNA)

>hsa_circ_0085534

TTTTTATTTAAGTACATTTTGCTTTTTAAAGTTGATTTTTTTCTATTGTTTTTAGAAAAAATAAAATAACTGGCAAATATATCATTGAGCCAAATCTTAAGACCCCCGAGCTGTGCTGCTCGCGGCCGCCACCGCCGGGCCCCGGCCGTCCCTGGCTCCCCTCCTGCCTCGAGAAGGGCAGGGCTTCTCAGAGGCTTGGC

**hsa_circ_0085535**

Divergent primer: GGCAAATATATCATTGAGCCAAA (left)

CCTCCTCGTCGCAGTAGAAA (right)

Junction sequence (3' End - 5' End of circRNA)

>hsa_circ_0085535

TTTTTATTTAAGTACATTTTGCTTTTTAAAGTTGATTTTTTTCTATTGTTTTTAGAAAAAATAAAATAACTGGCAAATATATCATTGAGCCAAATCTTAACAGCCTCCCGCGACGATGCCCCTCAACGTTAGCTTCACCAACAGGAACTATGACCTCGACTACGACTCGGTGCAGCCGTATTTCTACTGCGACGAGGAGG

**hsa_circ_0039407**

Divergent primer: ACGACCGCGACAAGAAGTAT (left)

GAGTCTCCCCCAACACCAGT (right)

Junction sequence (3' End - 5' End of circRNA)

>hsa_circ_0039407

GACAGCTGCACCACTGAGGGCCGCACGGATGGCTACCGCTGGTGCGGCACCACTGAGGACTACGACCGCGACAAGAAGTATGGCTTCTGCCCTGAGACCGAGCATGGCGATGGATACCCCTTTGACGGTAAGGACGGACTCCTGGCTCATGCCTTCGCCCCAGGCACTGGTGTTGGGGGAGACTCCCATTTTGATGACGA

**hsa_circ_0039408**

Divergent primer: TGACGGAAAGATGTGGTGTG (left)

CATAGGATGTGCCCTGGAAG (right)

Junction sequence (3' End - 5' End of circRNA)

>hsa_circ_0039408

GAGAGCTGCACCAGCGCCGGCCGCAGTGACGGAAAGATGTGGTGTGCGACCACAGCCAACTACGATGATGACCGCAAGTGGGGCTTCTGCCCTGACCAAGCCCTGTTCACCATGGGCGGCAACGCTGAAGGACAGCCCTGCAAGTTTCCATTCCGCTTCCAGGGCACATCCTATGACAGCTGCACCACTGAGGGCCGCAC

**hsa_circ_0039409**

Divergent primer: TGACGGAAAGATGTGGTGTG (left)

ATTTGTTGCCCAGGAAAGTG (right)

Junction sequence (3' End - 5' End of circRNA)

>hsa_circ_0039409

GCGCCGGCCGCAGTGACGGAAAGATGTGGTGTGCGACCACAGCCAACTACGATGATGACCGCAAGTGGGGCTTCTGCCCTGACCAAGCCATGTCCACTGTTGGTGGGAACTCAGAAGGTGCCCCCTGTGTCTTCCCCTTCACTTTCCTGGGCAACAAATATGAGAGCTGCACCA

**hsa_circ_0039410**

Divergent primer: TATTTGATGGCATCGCTCAG (left)

ATTTGTTGCCCAGGAAAGTG (right)

Junction sequence (3' End - 5' End of circRNA)

>hsa_circ_0039410

CCCCCACGCTGGGCCCTGTCACTCCTGAGATCTGCAAACAGGACATTGTATTTGATGGCATCGCTCAGATCCGTGGTGAGATCTTCTTCTTCAAGGACCGCCATGTCCACTGTTGGTGGGAACTCAGAAGGTGCCCCCTGTGTCTTCCCCTTCACTTTCCTGGGCAACAAATATGAGAGCTGCACCAGCGCCGGCCGCAG

**hsa_circ_0039411**

Divergent primer: CAAGGCACTTTTCACGTGTC (left)

ATTTGTTGCCCAGGAAAGTG (right)

Junction sequence (3' End - 5' End of circRNA)

>hsa_circ_0039411

CTCAATTAATAGAGTGCTTTCTATGTGCAAGGCACTTTTCACGTGTCACCTATTTTAACCTTTCCAACCACATAAATAAAAAAGGCCATTATTAGTTGAACCATGTCCACTGTTGGTGGGAACTCAGAAGGTGCCCCCTGTGTCTTCCCCTTCACTTTCCTGGGCAACAAATATGAGAGCTGCACCAGCGCCGGCCGCAG

**hsa_circ_0105604**

Divergent primer: CAGAGATGGGCATTGTCTCA (left)

CCCCTGCTAAATTCCTTGCT (right)

Junction sequence (3' End - 5' End of circRNA)

>hsa_circ_0105604

CACCAGAGATGGGCATTGTCTCAGCCCAGGTGGGCGGGTCTGGGTAGGTGGGTGTCTGGGGGGCAGTGTCTATGCTTGAAGTGGGTGAGGTCTCAGGAAGGTGGAGAATCCCACCTTTAAGCTGGTAAAATCAGACTGTGTCCTAGCAAGGAATTTAGCAGGGGATAAGAAAACCTCCTTTGGCCAGCCTCTGTCCACTG

**FOXM1**

Primer sequences:

Forward CCTCTCTCCCCGCATCAATC

Reverse CCGGCGCCAATTTCAAACA

Original sequences:

>NC_000012.12:2876299-2877165 Homo sapiens chromosome 12, GRCh38.p13 Primary Assembly

CCTCTCTCCCCGCATCAATCAGTAGATATTATTCCTCTTTTTCCACAGGATATCTAAGTCTTAATTTTCTCTTCTACTTCCTGACCAGCCCGTTATTATTATTGCCAACACTTTCTTGCCCGGGAAACTTCCAGAGTGGCTCCTCCGACCCATCCCCATCCTGAGCGGGGAAATCTTGCTACATTCTCAAGAACCAGGAAAAGGGCCCTGCAAAAGGGTAAGTAAGATGGAGGCGGTGTTGCCTAGTGGTGAGACAGCCTGGGAGCAGGGGAGTGTGTATGCAGGTTGGTCTTTGACTCAATGTAGTACAATCTCAGTCTTCATCTCAGCCACAGTCGGCCTAAGAAGGTAGGCCACCCCAGCCCTCCCGAGGCCGCGGGCAGGGTGCGGAGGTCAGACTTCCAGGGAAGGCCAAGGGGCGCTTTAAGCAGTGAGAAGGCCACGGCCAGGAGGTGGACGCAGAAAAAACTTGCACCCTGGCTCAGATTCTTCCCGTGTGACCCAAATCTCCACTCGTGGCCCGTGCCCTGCTGCTAGACGCCCTGACCTCCGCGCCCGGAGGCCTTGCTCCCCGCCTGTGCCCAGGCCAGGCCTGGGACTCCATTGCTGCATCCCGCTCACCTCCAGACTGCAGTCGCCGCCGCCGTTAGGCCGTAGCTCCGAAGGCGGGCTCCGGGCTCCTCCAACCTGGGGGCCGAGCCAGGGCCCCGGACGGGGGCTCGCGCCGGACCGGCCGGGTCCCCGGCGGTGCGGGCGGGGTGGGAACCCCGGGGGATCCCGGGAGGGGAGGGGGTCCCGGGCCGGGGCCGGGGGTGGGGTCTGGCACCGGAGCTTTCAGTTTGTTCCGCTGTTTGAAATTGGCGCCGG

**IGF2BP3**

Primer sequences:

Forward ACAACCTGGAAGCTGCCTTT

Reverse CTGGGGCTTCACTCAGAAGTT

Original sequences:

>NC_000007.14:23351008-23351710 Homo sapiens chromosome 7, GRCh38.p13 Primary Assembly

ACAACCTGGAAGCTGCCTTTCTGTCTTCCAGAGCTTCCCCAACCAGAAGAGGATTAGGTATCATTTGTTTTCCATTCATGTGTGAAAATCTGACGTTCCCTTTGAGAATGAGGATTAGCTCCAGATTTATATGAAATGAACACCAAGATACTGCAAGATTTTTACACATTCCCAAGTACTGTACAAGGAGTCCTCATGGCTGTGTTCAGAAGGGCACCAAGTACCAGAACGACACACTGTTACTAAGATTCCAAGGGGAATTCTCATGAACACCCACCACACACTCAGCATACTCACTTAGACTGGGTCTGTTTGGTGATGTTCCGAATGGTGGCACCTTCTTTTCCTATGATGGCTCCAACAAATTGGGTGGGAACCAGCAGGCGCAGAGGCAAATCACATGGTTTCTGCTTGGATACGGATCCTGGAGACCCCTGCCTTGAGGAGCCCCTCTGCCCAAGCCCCCGGCGACCTCGGGGCTGCTGCAAGGGGTTTTGCTGGGCGGCCATTTCATCAGGGATATAGGCTACTTTCAAGGTGAAATTCTCTAACTGAAATCCATTCAGTTTGTCTAGTGCTCTGAAAGTTGAAAAGGGGCAGGGGTGGGAAAAGGAATCAGGCTATATGACACATCTTTTAGCAAAGCAACACCCATCTCTTCTACTCAGCATTACTAGCTCTAAACTTCTGAGTGAAGCCCCAG

**GAPDH**

Primer sequences:

Forward GCAACTAGGATGGTGTGGCT

Reverse TCCCATTCCCCAGCTCTCATA

Original sequences:

>NC_000012.12:6536831-6537412 Homo sapiens chromosome 12, GRCh38.p13 Primary Assembly

GCAACTAGGATGGTGTGGCTCCCTTGGGTATATGGTAACCTTGTGTCCCTCAATATGGTCCTGTCCCCATCTCCCCCCCACCCCCATAGGCGAGATCCCTCCAAAATCAAGTGGGGCGATGCTGGCGCTGAGTACGTCGTGGAGTCCACTGGCGTCTTCACCACCATGGAGAAGGCTGGGGTGAGTGCAGGAGGGCCCGCGGGAGGGGAAGCTGACTCAGCCCTGCAAAGGCAGGACCCGGGTTCATAACTGTCTGCTTCTCTGCTGTAGGCTCATTTGCAGGGGGGAGCCAAAAGGGTCATCATCTCTGCCCCCTCTGCTGATGCCCCCATGTTCGTCATGGGTGTGAACCATGAGAAGTATGACAACAGCCTCAAGATCATCAGGTGAGGAAGGCAGGGCCCGTGGAGAAGCGGCCAGCCTGGCACCCTATGGACACGCTCCCCTGACTTGCGCCCCGCTCCCTCTTTCTTTGCAGCAATGCCTCCTGCACCACCAACTGCTTAGCACCCCTGGCCAAGGTCATCCATGACAACTTTGGTATCGTGGAAGGACTCATGGTATGAGAGCTGGGGAATGGGA
